# Genome assembly and characterization of a complex zfBED-NLR gene-containing disease resistance locus in Carolina Gold Select rice with Nanopore sequencing

**DOI:** 10.1371/journal.pgen.1008571

**Published:** 2020-01-27

**Authors:** Andrew C. Read, Matthew J. Moscou, Aleksey V. Zimin, Geo Pertea, Rachel S. Meyer, Michael D. Purugganan, Jan E. Leach, Lindsay R. Triplett, Steven L. Salzberg, Adam J. Bogdanove

**Affiliations:** 1 Plant Pathology and Plant Microbe Biology Section, School of Integrative Plant Science, Cornell University, Ithaca, NY, United States of America; 2 The Sainsbury Laboratory, University of East Anglia, Norwich, United Kingdom; 3 Center for Computational Biology, Johns Hopkins University, Baltimore, MD, United States of America; 4 Center for Genomics and Systems Biology, New York University, New York, NY, United States of America; 5 Center for Genomics and Biology, New York University Abu Dhabi, Saadiyat Island, Abu Dhabi, United Arab Emirates; 6 Department of Bioagricultural Sciences and Pest Management, Colorado State University, Fort Collins, CO, United States of America; 7 Departments of Biomedical Engineering, Computer Science, and Biostatistics, Johns Hopkins University, Baltimore, MD, United States of America; University of California Davis, UNITED STATES

## Abstract

Long-read sequencing facilitates assembly of complex genomic regions. In plants, loci containing nucleotide-binding, leucine-rich repeat (NLR) disease resistance genes are an important example of such regions. NLR genes constitute one of the largest gene families in plants and are often clustered, evolving via duplication, contraction, and transposition. We recently mapped the *Xo1* locus for resistance to bacterial blight and bacterial leaf streak, found in the American heirloom rice variety Carolina Gold Select, to a region that in the Nipponbare reference genome is NLR gene-rich. Here, toward identification of the *Xo1* gene, we combined Nanopore and Illumina reads and generated a high-quality Carolina Gold Select genome assembly. We identified 529 complete or partial NLR genes and discovered, relative to Nipponbare, an expansion of NLR genes at the *Xo1* locus. One of these has high sequence similarity to the cloned, functionally similar *Xa1* gene. Both harbor an integrated zfBED domain, and the repeats within each protein are nearly perfect. Across diverse Oryzeae, we identified two sub-clades of NLR genes with these features, varying in the presence of the zfBED domain and the number of repeats. The Carolina Gold Select genome assembly also uncovered at the *Xo1* locus a rice blast resistance gene and a gene encoding a polyphenol oxidase (PPO). PPO activity has been used as a marker for blast resistance at the locus in some varieties; however, the Carolina Gold Select sequence revealed a loss-of-function mutation in the PPO gene that breaks this association. Our results demonstrate that whole genome sequencing combining Nanopore and Illumina reads effectively resolves NLR gene loci. Our identification of an *Xo1* candidate is an important step toward mechanistic characterization, including the role(s) of the zfBED domain. Finally, the Carolina Gold Select genome assembly will facilitate identification of other useful traits in this historically important variety.

## Author summary

Plants lack adaptive immunity, and instead contain repeat-rich, disease resistance genes that evolve rapidly through duplication, recombination, and transposition. The number, variation, and often clustered arrangement of these genes make them challenging to sequence and catalog. The US heirloom rice variety Carolina Gold Select has resistance to two important bacterial diseases. Toward identifying the responsible gene(s), we combined long- and short-read sequencing technologies to assemble the whole genome and identify the resistance gene repertoire. We previously narrowed the location of the gene(s) to a region on chromosome four. The region in Carolina Gold Select is larger than in the rice reference genome (Nipponbare) and contains twice as many resistance genes. One shares unusual features with a known bacterial disease resistance gene, suggesting that it confers the resistance. Across diverse varieties and related species, we identified two widely-distributed groups of such genes. The results are an important step toward mechanistic characterization and deployment of the bacterial disease resistance. The genome assembly also identified a resistance gene for a fungal disease and predicted a marker phenotype used in breeding for resistance. Thus, the Carolina Gold Select genome assembly can be expected to aid in the identification and deployment of other valuable traits.

## Introduction

Recent advances in sequencing technology enable the assembly of complex genomic loci by generating read lengths long enough to resolve repetitive regions [1]. Repetitive regions are often hotspots of recombination and other genomic changes, but difficulties assembling them mean that they often remain as incomplete gaps for many years after a genome’s initial draft assembly. For example, the centromeres and telomeres remain unsequenced for nearly all plant and animal genomes today. The most straightforward way to span lengthy or complex repeats is to generate single reads that are longer than the repeats themselves, so that repeats can be placed in the correct genomic location. When repeats occur in tandem arrays, reads need to be longer than the entire array if one is to accurately determine the number of repeat copies that the array contains. One of the most promising current technologies for resolving complex repeats is nanopore-based sequencing from Oxford Nanopore Technologies ("Nanopore"), for which validated reads as long as 2,272,580 bases have been reported [2], and improvements in base calling software are increasingly improving fidelity [3]. Nanopore sequencing has been used for various applications, including genome sequencing of *Arabidopsis* and a wild tomato relative [4,5], resolving complex T-DNA insertions [6], and disease resistance gene enrichment sequencing [7].

Plant disease resistance loci represent an important example of complex portions of a genome that can be challenging to characterize in context using short-read sequencing. These loci often contain clusters of nucleotide binding leucine-rich repeat (NLR) protein genes. NLR proteins are structurally modular, typically containing an N-terminal coiled-coil (CC) domain or a Toll/interleukin-1 receptor (TIR) domain, a conserved nucleotide binding domain (NB-ARC), and a C-terminal region comprising a variable number of leucine-rich repeats (LRRs). The NLR gene family is one of the largest and most diverse in plants [8,9], with 95, 151, and 458 members reported in the reference maize, *Arabidopsis*, and rice genomes, respectively [10,11]. Fifty-one percent of the NLR genes occur in 44 clusters in the rice reference genome [12]. Plants lack an adaptive immune system, and it has been theorized that this clustering provides plants an arsenal of resistance genes that can rapidly evolve, through duplication and recombination, to respond to dynamic pathogen populations [13–16]. Indeed, the structure and content of NLR loci is variable, even in closely related cultivars, and, among plant populations, NLR genes account for the majority of copy-number and presence/absence polymorphisms [17–21]. Adding to the complexity of NLR genes, and the challenge of their sequence assembly, is the recent observation that approximately 10% of NLR genes encode additional, non-canonical, integrated domains (IDs) that may act as decoys, have roles in oligomerization or downstream signaling [22,23], or serve other functions. Analysis of closely related species has shown that these IDs appear to be modular, with independent integrations occurring in diverse NLR genes over evolutionary time [24].

In this study, we used Nanopore long reads combined with Illumina short reads to generate a high quality, whole genome assembly of rice cultivar Carolina Gold Select. Using the assembly we sought to delineate NLR gene content with a focus on a disease resistance locus, *Xo1*, which we identified in this variety in 2016 [25].

Carolina Gold Select is a purified line of Carolina Gold, a long-grain variety known for its distinctive gold hull and nutty flavor. Carolina Gold was the dominant variety grown in colonial America and is rumored to have been imported from Madagascar in 1685. It is a breeding ancestor of many modern US varieties [26]. Field production in the Carolinas stopped in 1927. McClung and Fjellstrom used trait data and molecular markers to produce the genetically uniform modern variety Carolina Gold Select in 2010 [26].

Genotyping and draft genome sequencing of Carolina Gold Select confirmed it to be in the tropical Japonica clade [27,28], but have not been sufficient to resolve loci associated with important disease resistance phenotypes, such as *Xo1*. *Xo1* protects against two important bacterial diseases, bacterial leaf streak (BLS) and bacterial blight (BB), caused by *Xanthomonas oryzae* pv. oryzicola (Xoc) and *X*. *oryzae* pv. oryzae (Xoo), respectively. It maps to a 1.09 Mb region of the long arm of chromosome four and segregates as a single dominant locus [25]. The *Xo1* locus overlaps several mapped loci for resistance to BB, including *Xa1*, *Xa2*, *Xa12*, *Xa14*, *Xa17*, *Xa31(t)*, and *Xa38*, that have been isolated from various rice cultivars [29–36]. Of these, only *Xa1* has been cloned, and it encodes an NLR with an N-terminal, integrated zinc-finger BED [zfBED; 37] domain and highly conserved, tandem repeats in the LRR region [38].

Though the molecular mechanism is not yet known, *Xo1* resistance is elicited by any of the ~20 transcription activator-like (TAL) effectors injected into the plant cell by any given Xoc or Xoo strain. TAL effectors are modular, type III-secreted, sequence-specific DNA binding proteins that directly transcriptionally activate host genes (see [39] for a review). They each are made up of an N-terminal type III secretion signal, a central DNA binding domain, and, in the C-terminal region, nuclear localization signals and an acidic activation domain. TAL effectors differ from one another, within and often across strains, in their DNA-binding specificity and the gene(s) they activate. Several have been identified that activate host genes that contribute to disease development [40]. A given TAL effector may trigger host resistance if it transcriptionally activates a so-called "executor resistance gene" [41]. Such genes are distinct from NLR genes, and their expression alone is sufficient for death of the host cell and effective defense against further invasion by the pathogen. Resistance triggered by a TAL effector independent of its ability to activate a gene is rarer, reported to date only for the pepper TIR-NLR protein Bs4 [42] and, more recently, the products of the *Xo1* locus and *Xa1* [25,43]. Interestingly, TAL effector-triggered, *Xo1*- and *Xa1*-mediated resistance is suppressed by pathogen delivery of N- and C-terminally truncated TAL effector proteins, called truncTALEs or iTALs, that are found (so far) exclusively in Asian strains of Xoo and Xoc [25,43,44].

Based on the functional similarity of *Xo1* to *Xa1*, and to *Bs4*, and the fact that the region corresponding to the *Xo1* locus in the rice reference genome (IRGSP-1.0; cv. Nipponbare, which lacks the BLS and BB resistance) [45] contains an array of seven NLR genes similar to each other (suggesting the potential for rapid evolution), we hypothesized that *Xo1*-mediated resistance in Carolina Gold Select is conferred by an NLR gene at the *Xo1* locus. The Carolina Gold Select genome assembly revealed fourteen such genes at the locus, including a candidate highly similar but not identical to *Xa1*, encoding an N-terminal, integrated zfBED domain and highly conserved, C-terminal, tandem repeats. In addition to the whole genome assembly, we present a detailed structural and comparative analysis of the *Xo1* candidate and other NLR genes at the *Xo1* locus, and an examination of zfBED-NLR gene content overall across representative species in the tribe Oryzeae.

## Results and discussion

### Carolina Gold Select genome assembly and annotation

To generate an assembly made up of large contigs with low error-rate, several assembly methods were used. We found that assembly by Flye [46] using only Nanopore data yielded long contigs but a high consensus error rate. MaSuRCA [47] assembly using both Illumina and Nanopore reads contained more sequence and had a very low consensus error rate, less than 1 error per 10,000 bases. We assessed the completeness of each assembly by aligning it to the Nipponbare reference genome using nucmer [48] with default parameters. The Flye and MaSuRCA assemblies covered 92% and 93% of the reference respectively. Combining the two assemblies resulted in a reconciled Carolina Gold Select assembly that benefited from both the higher quality consensus sequence and completeness of the MaSuRCA assembly, and the greater contiguity of the Flye assembly. Table 1 lists the quantitative statistics of both assemblies as well as the reconciled assembly. For N50 computations, we used a genome size estimate of 377,689,190 bp, equal to the total size of scaffolds of the final reconciled assembly.

**Table 1:** Quantitative statistics of Carolina Gold Select rice initial assemblies and the final reconciled assembly.

Assembly	N50 Contig^a^	N50 Scaffold	Output Sequence	# of contigs	# of scaffolds	Consensus error rate (errors per 10kb)
**MaSuRCA** (Illumina+Nanopore)	565,857	565,857	385,480,701	1,942	1,942	<1
**Flye** (Nanopore only)	1,492,039	1,497,653	362,619,590	649	634	142
**Reconciled Assembly**	1,632,109	1,719,775	377,688,090	1,297	1,286	7

^a^ Scaffold size of the final assembly (377,689,190 bp) used as genome size for N50 computations.

We found that the Carolina Gold Select assembly aligned to the Nipponbare reference genome with an average identity of 98.96%. 350,765,472 bases of the assembly (93%) aligned to 347,609,898 bases (93%) of the reference. The chromosome scaffolding process found 29 breaks in the scaffolds that were apparent mis-assemblies, and these were resolved. We call the final chromosomes Carolina_Gold_Select_1.0. The length statistics are provided in Table 2.

**Table 2:** Chromosome sizes for final Carolina Gold Select assembly.

Chromosome	Base pairs	Number of contigs
1	43,693,361	82
2	33,403,981	33
3	36,226,658	45
4	26,997,489	60
5	32,940,350	84
6	29,555,730	75
7	32,220,145	47
8	27,351,946	75
9	22,079,432	49
10	26,146,550	56
11	29,489,498	50
12	25,924,128	54
Unplaced	11,621,710	605

Protein coding genes were annotated based on the annotation of the reference genome (see Methods). For the 12 chromosomes, our mapping process identified and annotated 80,753 gene loci, of which 33,818 have protein coding transcripts. We identified a total of 86,983 transcripts, of which 40,047 are protein coding and have identified CDS features. The total number of bases covered by exons is 52,082,180 bp, or 14.2% of the total length of all 12 chromosomes, whose lengths sum to 366,055,270 bp.

### NLR genes in the Carolina Gold Select assembly

**Fig 1:**
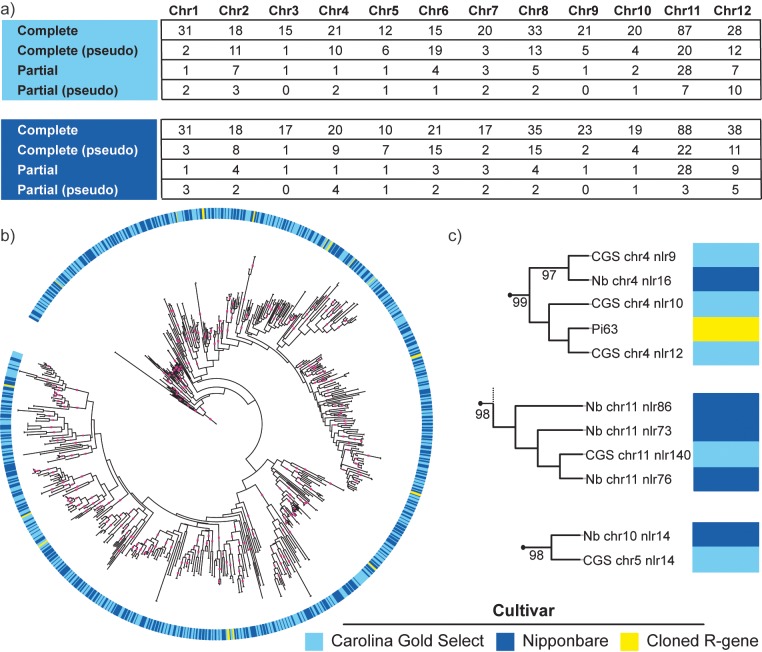
NLR proteins encoded in Carolina Gold Select in relation to Nipponbare and selected *R* genes. (a) Number and chromosomal distribution of all NLR-Annotator predicted NLR genes in Carolina Gold Select and Nipponbare assemblies. ‘Pseudo’, predicted NLR genes with stop codons in any domain. ‘Partial’, predicted NLR genes missing a canonical domain. All NLR gene types are included in order to provide a high-level comparison of NLR distribution in the two assemblies. (b) Maximum likelihood tree of encoded NB-ARC domains of NLR genes in Carolina Gold Select and Nipponbare, as predicted by NLR-Annotator. Incomplete NLR genes and genes with a stop codon in the NB-ARC domain are not included in the phylogeny. Sixteen cloned resistance genes are included for reference. Branches with bootstrap support greater than 80 percent are indicated with pink squares. Interactive tree available at http://itol.embl.de/shared/acr242. NB-ARC domain sequences available in S3 Table. (c) Examples of expansion (top), contraction (middle) and transposition (bottom) of NLR genes in Carolina Gold Select relative to Nipponbare. Bootstrap values greater than 80 percent are displayed. Further details available in S4 Table. In the example of expansion at the *Xo1* locus, as described in the text, CGS chr4 nlr9 is *CGS-Xo1*_*1*_, CGS chr4 nlr10 is *CGS-Xo1*_*2*_, CGS chr4 nlr12 is *CGS-Xo1*_*4*_, and Nb chr4 nlr16 is *Nb-xo1*_*1*_.

To identify NLR genes in the Carolina Gold Select genome, we used NLR-Annotator, an expanded version of the NLR-Parser tool [49]. NLR-Annotator does not rely on annotation data and does not mask repetitive regions, facilitating an unbiased analysis of the complete genome including NLR genes [50]. Because the NLR-Annotator pipeline has not been validated in rice, we first ran the pipeline on the well-annotated Nipponbare reference. A total of 518 complete or partial NLR genes were predicted. The list of 518 NLR genes is an overestimation of the number of true Nipponbare NLR genes in part because it includes complete and partial NLR genes, some of which are classified as pseudogenes due to the presence of a stop codon in a predicted coding sequence (Fig 1A). Genomic locations of the 518 Nipponbare NLR genes were cross-referenced with a list of 360 annotated Nipponbare NLR genes included in a recent analysis [24]; 356 matched. Of the four Nipponbare NLR genes that were not identified by NLR-Annotator, one lacks one or more canonical NLR gene domains based on InterProScan predictions. The other three appear to be complete, however, indicating an overall NLR-Annotator detection success rate of 99.2% (S1 Table). NLR-Annotator identified some complete NLR genes in the Nipponbare genome distinct from the 356; these may represent previously undetected NLR genes, pseudogenes, or false positives (highlighted in S1 Table).

Running the Carolina Gold Select assembly through the NLR-Annotator pipeline identified 529 total NLR genes. The Carolina Gold Select NLR genes are organized similarly to those of Nipponbare, occurring irregularly across the 12 chromosomes, with a large proportion occurring on chromosome 11 (Fig 1A and S2 Table). This similarity in number and genomic distribution of NLR genes provides support for the integrity of the Carolina Gold Select genome assembly.

To determine relationships between and among Nipponbare and Carolina Gold Select NLR genes, a maximum likelihood phylogenetic tree was generated using amino acid sequences of the central NB-ARC domain for all complete and complete pseudo- NLR genes, except those in which the NB-ARC domain is interrupted by a stop codon or has gaps greater than 50% across the alignment. NB-ARC domains from 16 cloned, NLR-type, rice resistance genes (S3 Table) were included to identify potential orthologs in Carolina Gold Select. Although the total number of predicted NLR genes is similar between the two cultivars, the resulting tree revealed 26 expansions and 37 contractions within NLR gene clades in Carolina Gold Select relative to Nipponbare, as well as 3 transpositions and 6 examples of combinations of transposition and either expansion or contraction (Fig 1B, Fig 1C, and S4 Table). Seven of the cloned resistance genes (*Pib*, *Pik2*, *Pi63*, *Pi2*, *RGA5*, *Pi36*, and *Pi37*) group with expanded or contracted NLR gene clades. The observed differences in NLR gene content in the two closely related cultivars is consistent with previous comparative analyses demonstrating that NLR gene families evolve rapidly and are characterized by presence-absence variation [19–21].

### Expansion at the Carolina Gold Select *Xo1* locus

**Fig 2:**
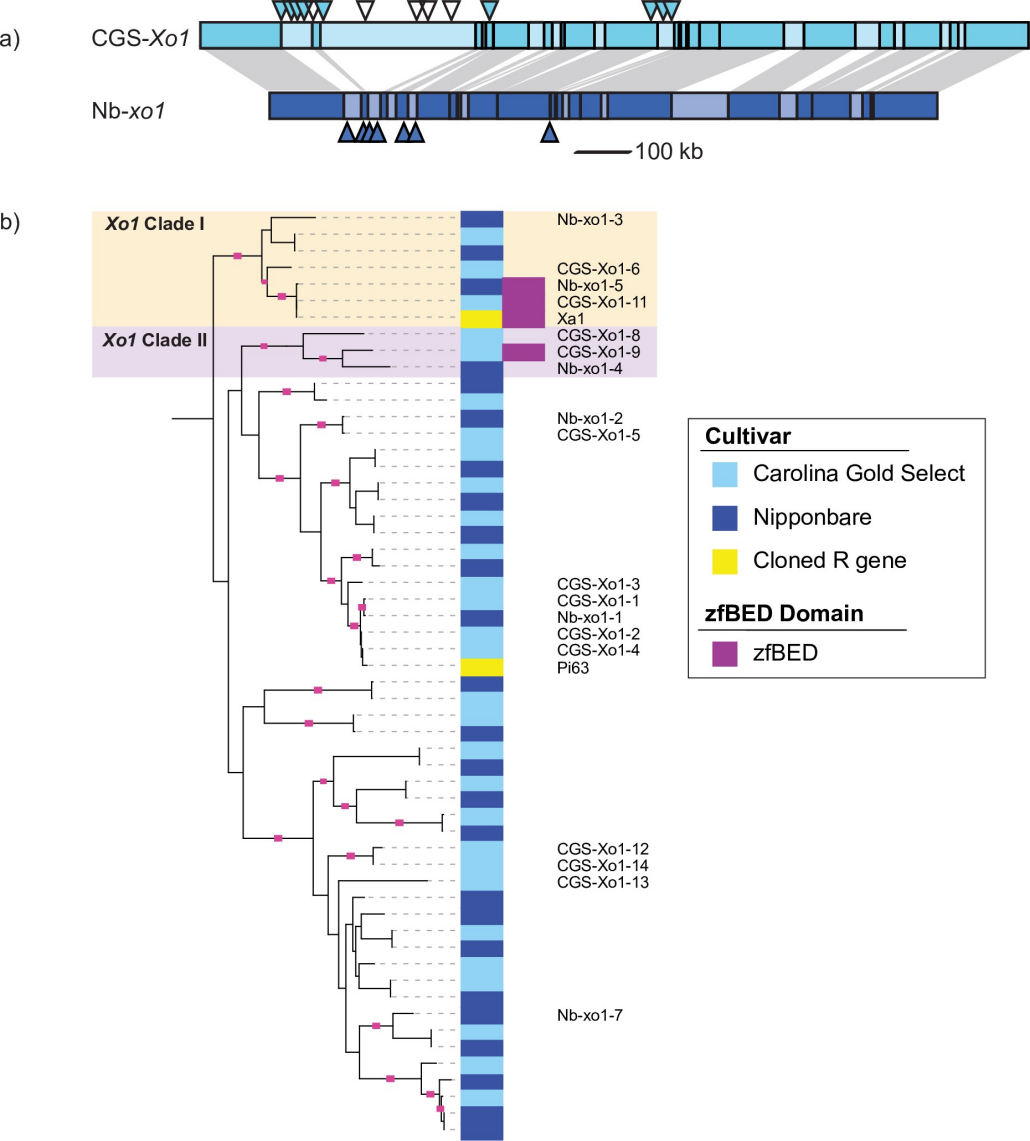
Expansion at the Carolina Gold Select *Xo1* locus and identification of an *Xo1* candidate. (a) Comparison of the *Xo1* locus in Carolina Gold Select and in Nipponbare. Areas of darker color on the two cartoon loci connected by gray shading represent regions of high similarity. Triangles indicate positions of NLR genes predicted by NLR-Annotator, designated from left to right as *CGS-Xo1*_*1*_ through *CGS-Xo1*_*14*_ in Carolina Gold Select and *Nb-xo1*_*1*_ through *Nb-xo1*_*7*_ in Nipponbare. Filled triangles indicate NLR genes expressed in leaf tissue during infection (see text and S9 Table). (b) An excerpt of the phylogenetic tree from Fig 1A containing the NLR genes at the *Xo1* locus and two known resistance genes, *Xa1* and *Pi63*. NLR genes encoding an integrated zfBED domain fall into two clades, which we designate as *Xo1* clades I and II. Branches with bootstrap support greater than 80 percent are indicated with pink squares. Interactive tree available at http://itol.embl.de/shared/acr242.

We extracted the region of the Carolina Gold Select assembly that corresponds to the 1.09 Mb Nipponbare *Xo1* mapping interval [25] and found that it spans a much larger region, 1.30 Mb, that includes a 182 kb insertion (Fig 2). It is unclear if this relative expansion is unique to a particular subgroup of *O*. *sativa* cultivars, but it is not present in the long-read (PacBio) assembly of *O*. *sativa* indica cultivar IR8 (S1 Fig) [51]. Hereafter, we refer to the region in Nipponbare, which as noted lacks the resistance to BLS and BB, as *Nb-xo1* and to the region in Carolina Gold Select as *CGS-Xo1*.

We mapped the NLR-Annotator output for Carolina Gold Select and Nipponbare onto the loci (Fig 2). There are 14 predicted NLR genes at *CGS-Xo1*, which we name *CGS-Xo1*_*1*_ through *CGS-Xo1*_*14*_ (*CGS-Xo1*_*1*,_ *CGS-Xo1*_*2*,_ *CGS-Xo1*_*4*,_ *CGS-Xo1*_*6*,_ and *CGS-Xo1*_*10*_ are predicted pseudogenes and *CGS-Xo1*_*15*_ is a predicted partial pseudogene). There are seven at *Nb-xo1*, matching the annotation of the reference genome; we refer to these as *Nb-xo1*_*1*_ through *Nb-xo1*_*7*_ (*Nb-xo1*_*1*_, *Nb-xo1*_*5*_, *Nb-xo1*_*6*_, and *Nb-xo1*_*7*_ are predicted pseudogenes). The NLR genes are not evenly distributed across the locus, but instead occur in clusters, consistent with the previous observation that only 24.1% of rice NLR genes occur as singletons [16].

### Identification of an *Xo1* candidate

Having delineated NLR gene content at the *Xo1* locus, we next sought to identify a candidate or candidates for the *Xo1* gene itself. First, using RNA sequencing (RNAseq), we asked which of the 14 predicted *CGS-Xo1* NLR genes are expressed in rice leaves following inoculation with an African strain of Xoc, that strain expressing a truncTALE, or a mock inoculum. The data provided evidence for expression of 9 of the 14 NLR genes including 4 of the 5 predicted pseudogenes (Fig 2). In contrast, each of the NLR genes at the locus in Nipponbare is expressed, based on previously obtained RNAseq data from leaves inoculated with the same African strain of Xoc [52]. The lack of expression data for nearly half the NLR genes at the *CGS-Xo1* locus led us to question whether the observed expansion at *CGS-Xo1* is an artifact of the assembly. To determine whether this is the case, we mapped all Nanopore reads to the assembly using BLASR [53], picked one best alignment for each read, and then examined the read coverage in the vicinity of the *CGS-Xo1* locus. The Nanopore reads covered the region with average depth of 21x, varying from 18x to 25x, providing robust support for the assembly. Thus, we considered the nine NLR genes expressed under the tested conditions to be candidates for *Xo1*; the other five may be non-functional, epigenetically silenced, or expressed under different conditions or tissues. We cannot rule out the possibility that the resistance is conferred by one or more of the non-NLR genes at the locus, but none of the annotations for those genes suggests a role in immunity (S5 Table).

**Fig 3:**
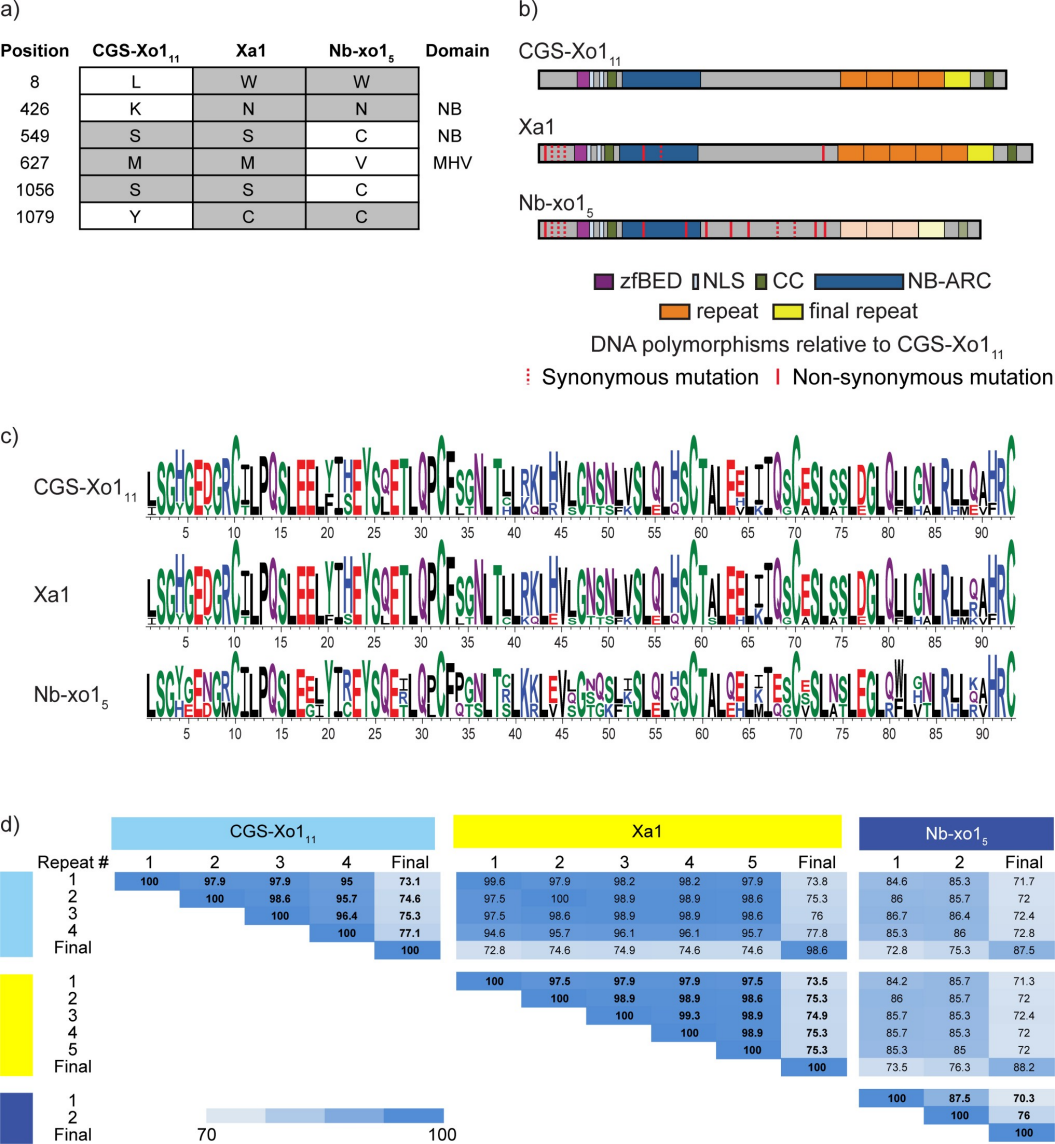
Structural comparison of the *Xo1* candidate *CGS-Xo1*_*11*_ with cloned *R* gene *Xa1* and with *Nb-xo1*_*5*_. (a) All amino acid polymorphisms upstream of the LRR in the three predicted gene products. (b) Cartoon alignment of predicted products of *CGS-Xo1*_*11*_, *Xa1*, and *Nb-xo1*_*5*_ showing the zfBED domains, nuclear localization signals (NLS), coiled coil domains (CC), NB-ARC domains, tandem repeats, and final repeats. Lighter shade of color for the repeats of *Nb-xo1*_*5*_ reflects their greater relative divergence. Synonymous and nonsynonymous nucleotide substitutions in relation to *CGS-Xo1*_*11*_ are indicated by dashed and solid red lines respectively. (c) WebLogos showing amino acid conservation of the tandem repeats in each LRR (d) Heatmap of repeat unit nucleotide sequence percent identity within and among the three coding sequences. *Nb-xo1*_*5*_ encodes an additional, cryptic repeat that does not align and is not included in (c) or (d).

Next, we inspected the NB-ARC domain-based phylogenetic tree and observed that the susceptible cultivar Nipponbare and the resistant cultivar Carolina Gold Select have one NLR gene each, *Nb-xo1*_*5*_ and *CGS-Xo1*_*11*_, that group closely with *Xa1*, the cloned BB resistance gene functionally similar to *Xo1* (Fig 2B). Several additional NLR proteins encoded at the *Nb-xo1* and *CGS-Xo1* loci fall into the same or a closely related clade. We call these *Xo1* clade I and *Xo1* clade II, respectively. They both reside in major integration clade (MIC) 3 defined by Bailey *et al*. [24]. Using the *Xa1* coding sequence as a guide, we extracted and aligned the corresponding sequences from *Nb-xo1*_*5*_ and *CGS-Xo1*_*11*_ (Fig 3). The MSU7 [45] gene model for *Nb-xo1*_*5*_ (LOC_Os04g53120) indicates that there is an intron downstream of the repeats; however, the sequence in the predicted intron aligns well to *CGS-Xo1*_*11*_ and *Xa1* coding sequence and therefore seems likely to be a mis-annotation. Thus, in our alignment we included it as coding sequence. Based on the Carolina Gold Select and Nipponbare genomic sequences, each of the coding sequences corresponds to three exons. The first is 307 bp and encodes no detectable, known protein domains. The second, 310 bp, encodes a non-canonical, integrated, 49 amino acid (aa) zfBED domain and a predicted, 9 aa nuclear localization signal (NLS). The third exon, the longest, encodes a second predicted 9 aa NLS, a 21 aa CC domain, a 288 aa NB-ARC domain, the LRR region, and a second, C-terminal, 21 aa CC domain. There are very few differences in the three genes upstream of the LRR-encoding region. In fact the zfBED domain, 2 NLSs, and first CC domain are 100% conserved at the nucleotide level. There is a single amino acid difference between the *CGS-Xo1*_*11*_ and *Xa1* NB-ARC domains, and two, distinct differences in that domain between *CGS-Xo1*_*11*_ and *Nb-xo1*_*5*_. In *Nb-xo1*_*5*_, the MHD triad, which has a role in NLR activation [54], has a M to V substitution. This substitution seems unlikely to be functionally relevant, however, as VHD has been observed in several functional CC-NLR proteins [55].

The LRR regions of *CGS-Xo1*_*11*_ and *Nb-xo1*_*5*_ share with *Xa1* the striking feature of highly conserved, tandem repeats. Though LRR regions are partially defined by their repetitive aa sequence, typically the repeats are polymorphic. The repeats within the *CGS-Xo1*_*11*_, *Nb-xo1*_*5*_, and *Xa1* LRR regions, each 93 aa (279 bp) in length, are nearly identical to one another. This near-identity of repeats is also found in alleles of the flax *L* and *M* resistance genes [56–58], though the repeat units of the *L* and *M* genes appear to have evolved independently: they are larger (~150 aa), and the genes belong to the Toll/interleukin-1 receptor class of NLR genes, which is not found in monocots. To explore this feature further, we analyzed all predicted NLR genes from the Nipponbare reference and the Carolina Gold Select assembly and found that, among the >1000 sequences, nearly identical LRRs are found only in NLR proteins encoded at the *Nb-xo1*/*CGS-Xo1* locus, though not all NLR genes there encode such repeats. *Xa1*, *CGS-Xo1*_*11*_, and *Nb-xo1*_*5*_, despite sharing the feature, differ in the number and conservation of their repeats. *Xa1* has five full repeats while *CGS-Xo1*_*11*_ has four and *Nb-xo1*_*5*_ three (Fig 3). Each gene encodes an additional, less conserved, final repeat. Intra- and inter-repeat comparison shows that *CGS-Xo1*_*11*_ and *Xa1* align well while *Nb-xo1*_*5*_ is more divergent (Fig 3). Overall, the sequence relationships suggest that *CGS-Xo1*_*11*_ is the *Xo1* gene. Functional analysis will be required to test this prediction definitively.

### *CGS-Xo1*_*11*_-like genes encoded in Oryzeae

**Fig 4:**
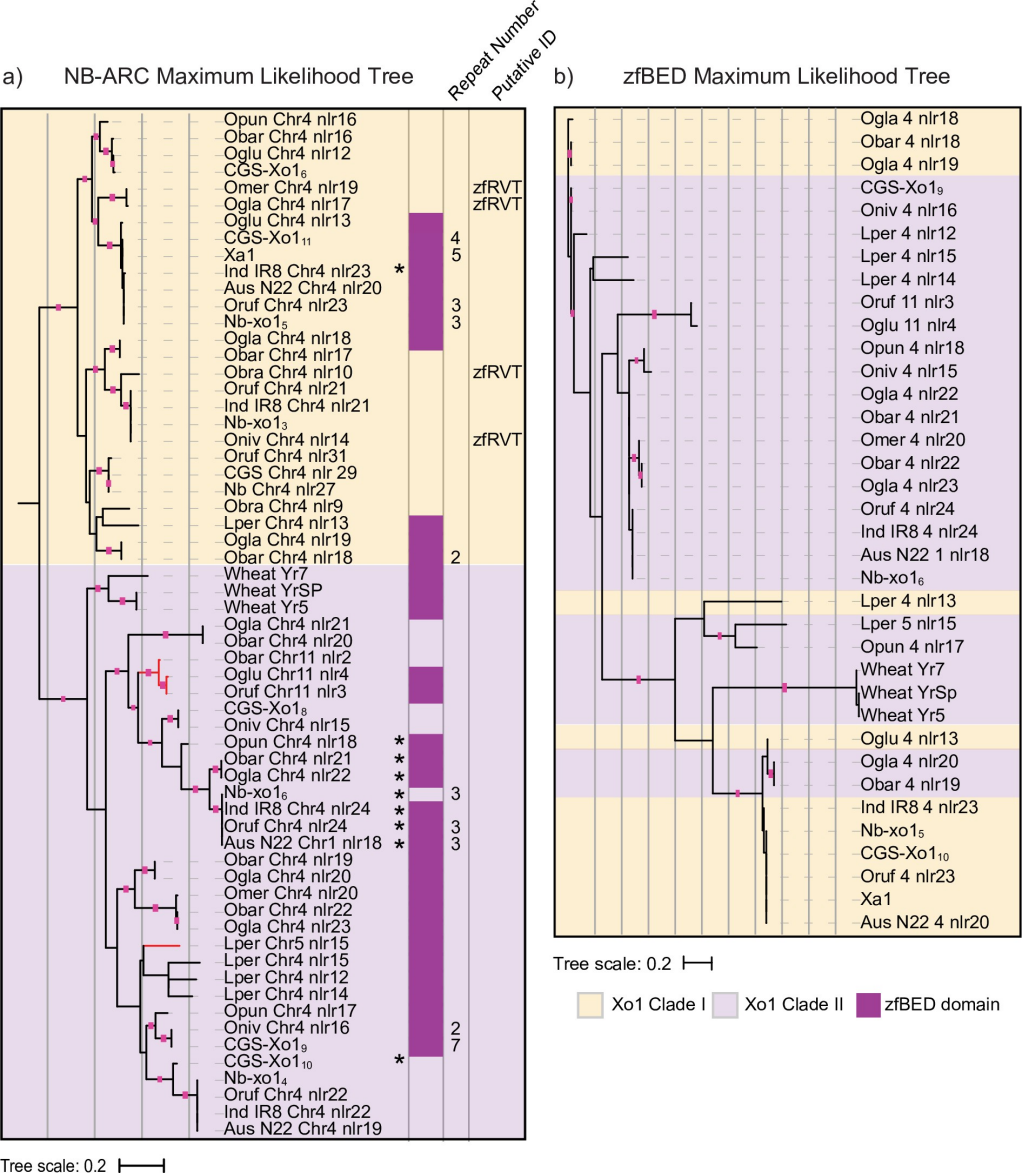
zfBED-NLR proteins across the Oryzeae. (a) *Xo1* clades I and II from an NB-ARC domain-based maximum likelihood tree of 5,078 predicted NLR proteins from representative Oryzeae genomes. Clade I proteins are indicated by orange shading, clade II by purple, and presence of zfBED domain by dark purple. Numbers of tandem 279 bp C-terminal repeats, where present, are given. Additional detected, non-canonical NLR gene motifs are noted. Red branches correspond to NLR genes not on chromosome four. Predicted NLRs with stops in the NB-ARC domain are annotated with asterisks. Full Oryzeae tree in S3 Fig and interactive tree available at http://itol.embl.de/shared/acr242. Nb Chr8 nlr 18 was used as an outgroup and can be viewed in the interactive tree. (b) Maximum likelihood tree of the 36 predicted Oryzeae zfBED-NLR proteins based on the zfBED domain amino acid sequence (zfBED sequences and nucleotide tree in S7 Table and S4 Fig). In a) and b), branches with bootstrap support greater than 80 percent are indicated with pink squares. Interactive trees available at http://itol.embl.de/shared/acr242.

The differences we observed in the presence of the zfBED domain and of the nearly identical repeats among NLR proteins encoded at the CGS-*Xo1* and Nb-*xo1* loci prompted us to characterize diversity of these features across the Oryzeae tribe. We ran the NLR-Annotator pipeline on the genomes of *Leersia perrieri*, *O*. *barthii*, *O*. *glaberrima*, *O*. *glumaepatula*, *O*. *brachyantha*, *O*. *meridinalis*, *O*. *nivara*, *O*. *punctata*, *O*. *rufipogon*, *O*. *sativa* IR8, and *O*. *sativa* Aus N22 [51,59,60]. All NB-ARC domains identified were added to those of Nipponbare and Carolina Gold Select. In this analysis, to capture the distribution of all zfBED and/or near-perfect repeat-containing genes, we chose to include NLR genes with stop codons. These >5,000 sequences were used to generate an Oryzeae NLR gene maximum likelihood phylogenetic tree (S2 Fig), without bootstrapping. Two distinct sister clades in the tree respectively include the previously identified Carolina Gold Select and Nipponbare *Xo1* clade I and II NLR genes. For all identified members of these clades, a new maximum likelihood tree was generated using the NB-ARC domains, with bootstrapping (Fig 4). Additionally, full sequences of the NLR genes represented in these expanded *Xo1* clades I and II were extracted and examined for the presence of a zfBED domain, other IDs, and nearly identical repeats (Fig 4 and S6 Table). Because several of the genomes were assembled from short-reads, it is likely that some of the NLR genes are misassembled. However, we made the following observations. NLR genes from each genome are found in each clade,though not all clade I and II NLR genes encode a zfBED domain, and no NLR genes of *O*. *brachyantha* do. Nearly identical repeats are found only in NLR genes with a zfBED domain, though there are several zfBED-NLR genes without them. A zfRVT domain (zinc-binding region of a putative reverse transcriptase; Pfam 13966) was predicted in four *Xo1* clade I Oryzeae NLR genes as well as one of the wheat Yr alleles from *Xo1* clade II. The zfRVT domain has been detected in previous NLR gene surveys [23]. Most of the NLR genes in the two clades reside in the *Xo1* locus on chromosome four, however there are six, all from *Xo1* clade II, that are on other chromosomes; this is consistent with research demonstrating that transposition events are common during evolution of NLR gene families [61].

The presence of closely related zfBED-NLR genes across diverse Oryzeae species suggests that the integration of the zfBED domain preceded Oryzeae radiation. This inference is consistent with a recent analysis that identified NLR genes encoding N-terminal zfBED domains in several monocot species including *Setaria italica*, *Brachypodium distachyon*, *Oryza sativa*, *Hordeum vulgare*, *Aegilops tauschii*, *Triticum urartu*, and *Triticum aestivum*, though no zfBED-NLR genes were detected in *Sorghum bicolor* or *Zea mays* [24]. ZfBED-NLR genes have also been detected in dicots, with as many as 32 reported in poplar (*Populus trichocarpa*) [62]. A more recent analysis that includes *P*. *trichocarpa* detected 26 zfBED-NLR genes, of which 24 have the same architecture as *CGS-Xo1*_*11*_, with the zfBED domain encoded upstream of the NB-ARC and LRR domains [23]. Nevertheless, it is unclear if all zfBED-NLR genes arose from a single integration, or if the integration has occurred independently in the monocot and dicot lineages. Distribution among dicots seems limited, and a recent delineation of the *Arabidopsis* pan ‘NLR-ome’ generated from 65 accessions found none [63].

Three alleles of a zfBED-NLR gene in wheat, *Yr5*, *Yr7*, and *YrSP*, were recently shown to provide resistance to different strains of the stripe rust pathogen, *Puccinia striiformis* f. sp. tritici [64]. The *Yr5*/*Yr7*/*YrSP* syntenic region in the Nipponbare genome, determined by the authors of that study, overlaps *Nb-Xo1*_*5*._ When added to the Oryzeae tree, the NB-ARC domains of the wheat rust resistance alleles group with *Xo1* clade II (Fig 4). It is remarkable that these evolutionarily-related NLR genes with similar non-canonical N-terminal fusions provide resistance to two pathogens from different kingdoms of life. In this context it is also worth noting that the poplar *MER* locus for *Melampsora larica-populina* rust resistance was reported to contain 20 of the 32 poplar zfBED-NLR genes [62].

It has been demonstrated in rice and *Arabidopsis* that IDs in NLR proteins can act as decoys for pathogen effector proteins such that their interaction with an effector activates the NLR protein and downstream defense signaling [23,65–67]. If this were the case for the zfBED domain, we might expect to see distinct signatures of evolution in the zfBED and NB-ARC domains. We extracted the zfBED domains from 33 Oryzeae zfBED-NLR genes as well as the three wheat *Yr* alleles and created a tree to determine if they would form two sub-groups, similarly to the NB-ARC domains. They do not, even when the tree is generated from the nucleotide sequences (Fig 4 and S7 Table and S3 Fig). The zfBED domain of *Xo1* clade I and II NLR genes thus appears to be under distinct selective pressures from the NB-ARC domain. Alternatively, the discordance between the NB-ARC and zfBED trees may be evidence of domain swapping, as has been reported for other integrated domain-encoding NLR genes [68].

The role or roles of the zfBED domain remain unclear. The domain was first identified in the *Drosophila* DNA binding proteins BEAF32A/B and DREF and, based on comparative phylogenetic analysis, is believed to have derived from a transposon and to have been co-opted by hosts on two or more occasions [37]. In plants, Aravind [37] identified the zfBED domain in DNA-binding proteins involved in light response and fruit ripening [69,70]. The zfBED domain of *Arabodpsis* transposase DAYSLEEPER was later shown experimentally to bind DNA specifically [71]. The observations that *Yr7*, *Yr5*, and *YrSP* have identical zfBED sequences but recognize different pathogen races [64] and that *Xa1*, *CGS-Xo1*_*11*_, and *Nb-xo1*_5_ encode identical zfBEDs do not support the model of this domain being a specificity-determining decoy. Rather, the domain may have a role in downstream signaling, a role in localization, or some other role. Mechanisms might include dimerization, recruitment of other interacting proteins, or DNA binding.

### The Carolina Gold Select *Xo1* locus contains a rice blast resistance gene

In the NB-ARC domain-based tree (Fig 1), *CGS-Xo1*_*2*_ and *CGS-Xo1*_*4*_ group with rice blast resistance gene *Pi63*, originally cloned from rice cultivar Kahei [72,73]. Direct sequence comparison revealed that *Xo1*_*4*_, which is expressed (Fig 2B), is *Pi63*: the genomic sequences, including 3 kb upstream of the gene bodies, are 100% identical. Modern US rice varieties, some of which descend from initial Carolina Gold populations, contain several blast resistance genes including *Pik-h*, *Pik-s*, *Pi-ta*, *Pib*, *Pid*, and *Pi2* [reviewed in 74], but each of these genes was introduced into the US germplasm from Asian cultivars, and none resides on chromosome four. Our discovery of *Pi63* in Carolina Gold Select reveals that this variety may be a useful genetic resource for further strengthening US rice blast resistance.

The presence of blast and blight resistance at the *Xo1* locus in Carolina Gold Select is reminiscent of *O*. *sativa* japonica cultivar Asominori. Asominori is the source of the blast resistance gene *PiAs(t)* and the BB resistance gene *Xa17*, and both of these genes, though not yet cloned, map to the *Xo1* region of chromosome four. *Xa17*, previously *Xa1-As(t)*, has a similar resistance profile to *Xa1* but provides resistance at both seedling and adult stages; *Xa1* is unstable at the seedling stage [32]. *PiAs(t)* and *Xa17* are closely linked to a polyphenol oxidase (PPO) gene, the activity of which can be detected by treating seeds with phenol [32]. This seed-treatment assay has been used as a surrogate to track the blight and blast resistance genes during crosses [31,32]. In the Carolina Gold Select genome assembly, *CGS-Xo1*_*4*_ (*Pi63*) and *CGS-Xo1*_*11*_ are separated by 270 kb, and a PPO gene resides an additional 175 kb downstream. However, the Carolina Gold Select PPO gene sequence has a 29 bp loss-of-function deletion common in japonica cultivars [75]. The seed treatment assay confirmed absence of PPO activity (S4 Fig). It seems likely that the genomic arrangement at the Asominori blight and blast resistance locus is similar to that in Carolina Gold Select, though with an intact PPO gene. Our results illustrate that while the seed treatment assay may be useful to track resistance at the *Xo1* locus in some cases, such as crosses with Asominori, in others it may not, due to a loss of function mutation in the linked PPO gene. More broadly, our results demonstrate the ability to make phenotypic predictions based on the Carolina Gold Select assembly.

### Conclusions

In this study, whole genome sequencing using Nanopore long reads along with Illumina short reads delineated a complex, NLR gene-rich region of interest, the *Xo1* locus for resistance to BLS and BB, in the American heirloom rice variety Carolina Gold Select. This revealed an expansion at the locus relative to the reference (Nipponbare) genome and allowed identification of an *Xo1* gene candidate based on sequence similarity to the functionally similar, cloned *Xa1* gene, including an intergrated zfBED domain and nearly identical repeats. Analysis of NLR gene content genome-wide and comparisons across representative members of the Oryzeae and other plant species identified two sub-clades of such NLR genes, varying in the presence of the zfBED domain and the number of repeats. These results support the conclusion of Bailey *et al*. [24] that the zfBED domain was integrated prior to the differentiation of the Oryzeae, possibly before divergence of monocots and eudicots. Additional analysis revealed that the zfBED domain has been under different selection from the NB-ARC domain. The results also provided further evidence that the zfBED domain can be identical not only among resistance alleles with different pathogen race specificities but also between resistance genes that recognize completely different pathogens [64]. Considering *CGS-Xo1*_*11*_ and *Nb-Xo1*_*4*_, the results also suggested that the zfBED domain can be identical between functional and non-functional, expressed resistance gene alleles. Finally, the genome sequence uncovered a known rice blast resistance gene at the *Xo1* locus and a loss of function mutation in a linked, PPO gene. The latter breaks the association of PPO activity with BB and blast resistance that has been the basis of a simple, seed staining assay for breeders to track the resistance genes in some crosses.

Our study illustrates the feasibility and benefits of high quality, whole genome sequencing using long- and short-read data to resolve and characterize individual, complex loci of interest. It can be done by small research groups at relatively low cost: our sequencing of the Carolina Gold Select genome used data generated from a single Illumina HiSeq2500 lane and two ONT MinION flowcells. Because long-read sequencing technologies and base-calling continue to improve, it seems likely that high quality assemblies from long-read data alone will become routine. The long-read data enabled us to identify and characterize the expansion of NLR genes at the *Xo1* locus. Such presence/absence variation across genotypes is hard if not impossible to determine definitively by only short-read sequencing. The long-read data, with short-read error correction, also allowed us to define the number and sequences of nearly identical repeats in the *Xo1* gene candidate *CGS-Xo1*_*11*_ and genes like it in Carolina Gold Select. Indeed, we caution that, in short-read assemblies, sequences of *CGS-Xo1*_*11*_ homologs and other such repeat-rich genes, or repeat-rich intergenic sequences, should be interpreted with care due to the possibility of artificially collapsed, expanded, or chimeric repeat regions.

Cataloging NLR gene diversity in plants is of interest for resistance gene discovery, for insight into NLR gene evolution, and for clues regarding the functions of IDs. Sequence capture by hybridization approaches, such as RenSeq, have been developed and applied to catalog NLR genes in representative varieties of several plant species [76–82], but these depend on *a priori* knowledge to design the capture probes and thus may miss structural variants. Also, they do not reveal genomic location, recent duplications, or arrangement of the genes, information necessary to investigate evolutionary patterns. Sequence capture of course also misses integrated domains or homologs encoded in non-NLR genes, precluding broader structure-function and evolutionary analyses. Sequence capture is nevertheless likely to continue to play an important role in organisms with large, polyploid, or otherwise challenging genomes.

The Carolina Gold Select genome sequence is among a still relatively small number of high quality assemblies for rice and the first of a tropical japonica variety. The identification of an *Xo1* candidate is a significant step toward cloning and functional characterization of this important gene and will facilitate investigation of the role(s) of the integrated zfBED domain in NLR gene-mediated resistance. The Carolina Gold Select genome assembly will be an enabling resource for geneticists and breeders to identify, characterize, and make use of genetic determinants of other traits of interest in this historically important rice variety.

## Materials and methods

### Genomic DNA extraction and Nanopore sequencing

Carolina Gold Select seedlings were grown in LC-1 soil mixture (Sungro) for three weeks in PGC15 growth chambers (Percival Scientific) in flooded trays with 12-hour, 28°C days and 12-hour, 25°C nights. Three weeks after planting leaf tissue was collected and snap frozen in liquid nitrogen.

Genomic DNA was extracted from 250 mg of frozen leaf tissue with the QIAGEN g20 column kit with 0.5 mg/ml cellulase included in the lysis buffer. Eluted DNA was cleaned up with 1 volume of AMPure XP beads (Beckman-Coulter). To attain the recommended ratio of molar DNA ends in the Nanopore library preparation, the genomic DNA was sheared with a Covaris g-TUBE for one minute at 3800 RCF on the Eppendorf 5415D centrifuge. A 0.7x volume of AMPure XP beads was used for a second clean-up step to remove small DNA fragments. Sheared DNA was analyzed on a NanoDrop spectrophotometer (Thermo Fisher) to determine A260/280 and A260/230 ratios, and quantified using the Qubit dsDNA BR (Broad Range) assay kit (ThermoFisher). Fragment length distribution was visualized with the AATI Fragment Analyzer (Agilent). Sheared genomic DNA was used as input into the Nanopore LSK108 1D-ligation library prep kit, then loaded and run on two R9.4.1 MinION flow cells. Raw reads for both flow cells were base-called with Albacore v2.3.0. Amounts of DNA at each step of the workflow can be found in S8 Table. Run metrics were calculated using scripts available at https://github.com/roblanf/minion_qc.

### Illumina sequencing

Genomic DNA was isolated from leaf tissue of a single Carolina Gold Select plant using the Qiagen DNEasy kit. Libraries were prepared as described [83], using the Illumina TruSeq kit with an insert size of ∼380 bp. Two × 100-bp paired-end sequencing was carried out on an Illumina HiSeq 2500.

### Sequence assembly

Reads were assembled using default settings with two different assembly programs, MaSuRCA version 3.2.7 [47] and Flye version 2.4.1 [46], followed by reconciliation of the results to produce an initial contig/scaffold assembly of the genome, CG_RICE_0.9. In reconciliation we followed the procedure described in [84]. We merged the contigs from the more contiguous Flye assembly with MaSuRCA contigs by mapping the assemblies to each other using Mummer4 with default parameters [48], and then filtering the alignments to select those longer than 5000 bp that were reciprocal best hits, using the delta-filter program from the Mummer 4 package with options “-l 5000–1”. We then looked for cases where a contig of the MaSuRCA assembly merged two contigs of the Flye assembly uniquely, overlapping the ends of two Flye contigs by 5000 bp or more on both sides of the merge. This resulted in longer merged contigs, although they still had the lower-quality consensus of the Flye assembly. We then used the “polish_with_illumina_assembly.sh” script from the MaSuRCA package to improve the consensus quality. The scripts first aligned the MaSuRCA assembly contigs to the merged contigs using Mummer4, then filtered for unique best alignments for each contig using “delta-filter -1”, and finally replaced the aligned sequence of the merged contigs with the aligned MaSuRCA sequence, resulting in a highly contiguous, merged assembly with low consensus error rate. Consensus error rate was computed using the script ‘evaluate_consensus_error_rate.sh’ distributed with MaSuRCA, which was created following Jain and colleagues [85]; this script maps the Illumina data to the assembly using bwa [86], and then calls short sequence variants using the FreeBayes software [87]. A sequence variant at a site in a contig sequence was considered an error if all Illumina reads disagree with the contig and there are at least three Illumina reads that agree on an alternative. Sequence variants found through this procedure included both SNPs and short insertions/deletions. The total number of bases in error variant calls was taken as the total number of errors, and the error rate was computed as total number of errors divided by the sequence size.

Following the completion of the assembly, we used the Nipponbare rice reference genome IRGSP-1.0 (NCBI accession GCF_001433935) [45] to order and orient the assembled scaffolds on the chromosomes using the MaSuRCA chromosome scaffolder tool, available as part of MaSuRCA v3.2.7 and later.

### Reference-based annotation

We annotated the 12 assembled chromosome sequences by aligning the transcripts from the rice annotation produced by the International Rice Genome Sequencing Project (IRGSP) and the Rice Annotation Project Database (RAP-DB) [45] We used release 1.0.40 of the annotation file for *Oryza sativa* made available by Ensembl Plants [88]. We aligned the DNA sequences of these transcripts to our assembled chromosomes using GMAP [89]. The resulting exon-intron mappings were further refined for transcripts annotated as protein coding, as follows. For each protein-coding transcript in our assembled chromosomes, we extracted the transcript sequence using gffread (http://ccb.jhu.edu/software/stringtie/gff.shtml) and aligned it with the protein sequence from the IRGSP annotation to identify the correct start and stop codon locations. These protein-to-transcript sequence alignments were performed using blat [90], followed by a custom script (https://github.com/gpertea/gscripts/tree/master/remap_ann) that projected the local CDS coordinates back to the exon mappings on our assembled chromosome sequences, to complete the annotation of the protein-coding transcripts.

### RNA extraction and sequencing

Three-week old Carolina Gold Select seedlings grown under the conditions described above were syringe-infiltrated with an OD_600_ 0.4 suspension of African Xoc strain CFBP7331 carrying a plasmid-borne copy of the truncTALE gene *tal2h* or empty vector [44], or mock inoculum (10 mM MgCl_2_). Each leaf was infiltrated at 20 contiguous spots starting at the leaf tip. Inoculated tissue was harvested 24-hours post-infiltration, before the hypersensitive reaction manifested for CFBP7331 with empty vector. The experiment was repeated three times. RNA was extracted from the replicates with the QIAeasy RNA extraction kit (Qiagen) and submitted to Novogene Biotech for standard, paired-end Illumina sequencing.

For Nipponbare, previously generated RNAseq data were used (Accessions SRX978730, SRX978731, SRX978732, SRX978723, SRX978722, and SRX978721, Short Read Archive of the National Center for Biotechnology Information). These data were generated from leaf tissue collected 48 hours after inoculation with CFBP7331 [52].

### NLR gene expression analysis

We used the ‘quant’ function in Salmon [91] to quantify expression of NLR genes in the Nipponbare and Carolina Gold RNAseq datasets referenced above. For each of the two varieties, genomic sequences of all NLR-Annotator-identified genes plus 1 kb upstream and 1kb downstream were extracted and used as indices. The additional sequences on each end were included in an effort to capture the entire transcript while avoiding transcripts for any genes encoded nearby. RNAseq reads were mapped to the respective NLR indices, and those genes with an average of >500 Transcripts per Kilobase Million, for any treatment or condition across replicates, were considered expressed (S9 Table).

### NLR gene identification and phylogenetic analysis

NLR gene signatures were detected with NLR-Annotator [92] using a sequence fragment length of 20 kb with 5 kb overlaps. NLR-Annotator predictions for Nipponbare were compared to previously annotated NLR genes using BED-tools intercept [93]. NLR-Annotator categorizes predicted NLR genes as ‘complete’, ‘complete (pseudogene)’, ‘partial’, or ‘partial (pseudogene)’. All NLR gene types are included in the chromosomal distribution shown in Fig 1C, however only complete NLR genes with less than 50% gaps in the alignment were used for any phylogenetic analyses. Trees were generated with RAxML v8.2.12 [94] with the MRE bootstrap parameter to determine sufficient bootstrapping for tree convergence. The best tree for each analysis was visualized using the Interactive Tree of Life (iTOL) tool [95], with bootstrap support shown. Detailed RAxML parameters including substitution models are available in S10 Table.

To generate the phylogeny of Carolina Gold Select, Nipponbare, and cloned *R* genes used for Fig 1 and Fig 2B, encoded NB-ARC amino acid sequences were identified and aligned using NLR-Annotator. NB-ARC domains of complete NLR genes that include a stop codon were excluded from the analysis at this point. Sequences for this and subsequent analyses are available in S3 Table.

The NLR-Annotator pipeline was repeated for the additional Oryzeae species, and aligned NB-ARC domains were added to those of Carolina Gold Select, Nipponbare, and the cloned *R* genes. As noted, sequences with greater than 50% missing data were excluded. A maximum likelihood tree was generated using this list of NB-ARC sequences, in this case including NB-ARC sequences with stop codons. This ‘all Oryzeae’ tree (S2 Fig) was not bootstrapped due to the size of the alignment. NB-ARC sequences identified in the ‘all Oryzeae’ tree that fell into *Xo1* clade I and II were extracted and reanalyzed with sufficient bootstraps to generate a converged tree (Fig 4A). The same method was also used for both the amino acid and nucleotide trees in Fig 4B and S3 Fig. The zfBED domain sequences are available in S7 Table, and RAxML details are available in S10 Table.

Integrated domains outside of the canonical NLR gene structure were detected by running the NLR-Annotator-identified genes plus the 5 kb 5′ and 5 kb 3′ flanking sequences (S11 Table) through Conserved Domain BLAST [96] using default parameters. Domains >2 kb from a known NLR gene domain were considered likely false positives and disregarded. Domains deemed likely to be annotations of LRR sub-types were also excluded.

### Tandem repeat characterization

Self-comparison dotplots were used to determine whether NLR-Annotator-identified genes in Nipponbare and Carolina Gold Select contain nearly identical repeats. To define repeat units in a standardized way, *Xo1* clade I and II NLR gene sequences were extracted and submitted to Tandem Repeats Finder with default parameters [97]. WebLogos for aligned repeats of *CGS-Xo1*_*11*_, *Xa1*, and *Nb-xo1*_*6*_ were generated using WebLogo3 [98].

## Supporting information

S1 Table NLR-Annotator output for Nipponbare cross-referenced with the MSU 7 annotation. (XLSX)

S2 Table NLR-Annotator output for Carolina Gold Select. (XLSX)

S3 Table NB-ARC domain sequences used to generate the phylogenetic tree in Fig 1. (TXT)

S4 Table Expansion, contraction, and transposition of NLR gene clades in Carolina Gold Select relative to Nipponbare. (XLSX)

S5 Table Annotated genes at the Carolina Gold Select *Xo1* locus. (XLSX)

S6 Table Integrated domains detected with CD BLAST. (XLSX)

S7 Table zfBED domain sequences from the zfBED-NLR genes across the Oryzeae represented in Fig 3. (DOCX)

S8 Table Nanopore DNA sequencing metrics. (XLSX)

S9 Table TPM values for Nipponbare and Carolina Gold Select NLR-Annotator predicted genes. (XLSX)

S10 Table Details for RaxML analysis. (TXT)

S11 Table NLR gene sequences plus 5 kb on either side including integrated domains. (FA)

S1 Fig Dotplot comparison of the *Xo1* locus in Carolina Gold Select and IR8. Dotplot comparison of the *Xo1* region of Carolina Gold Select (chr4 22729801..24027920) and indica cultivar IR8 (chr4 31657596..32688920). The yellow box highlights the insertion that is present in Carolina Gold Select and absent in IR8.
(PDF)

S2 Fig Maximum likelihood tree of NLR genes across Oryzeae. Maximum likelihood tree of 5,083 NB-ARC domain amino acid sequences detected by NLR-Annotator in representative Oryzeae genomes. Tree includes known rice *R*-genes and three wheat zfBED-NLRs. NB-ARC domains with stop codons were included in the tree. *Xo1* clade I and II are highlighted with orange and purple branches respectively. NB-ARC amino acid sequences are available in Supplemental S3 Table. Tree file is available at iTOL– http://itol.eml.de/shared/acr242.
(PDF)

S3 Fig Maximum likelihood tree of *Xo1* zfBED nucleotide sequences. Maximum likelihood tree of zfBED domain nucleotide sequences from Xo1 clade I and II NLRs. Branches with bootstrap support greater than 80 percent are indicated with pink squares. Interactive tree available at http://itol.embl.de/shared/acr242.
(PDF)

S4 Fig Polymorphism at the polyphenol oxidase gene linked to BB and blast resistance genes at the *Xo1* locus. (a) Amino acid sequence alignment of *Oryza sativa* indica cultivar MH63 functional polyphenol oxidase (PPO) gene with the sequence of Carolina Gold Select PPO. Loss-of-function mutation highlighted in yellow. (b) Polyphenol oxidase activity in seeds of the known PPO positive rice cultivar Asominori and Carolina Gold Select with and without hull. Dark brown indicates activity.
(PDF)
