# Supplementary material for: Genome assembly and characterization of a complex zfBED-NLR gene-containing disease resistance locus in Carolina Gold Select rice with Nanopore sequencing
Source: PLoS Genet. 2020 Jan 27;16(1):e1008571. doi: 10.1371/journal.pgen.1008571 (PMC7004385; doi:10.1371/journal.pgen.1008571)
Supplement: S1 Fig — Dotplot comparison of the Xo1 region of Carolina Gold Select (chr4 22729801..24027920) and indica cultivar IR8 (chr4 31657596..32688920). The yellow box highlights the insertion that is present in Carolina Gold Select and absent in IR8. (PDF) [file pgen.1008571.s012.pdf]

S1 Figure

Dotplot comparison of the *Xo1* locus in Carolina Gold Select and Indica cultivar IR8

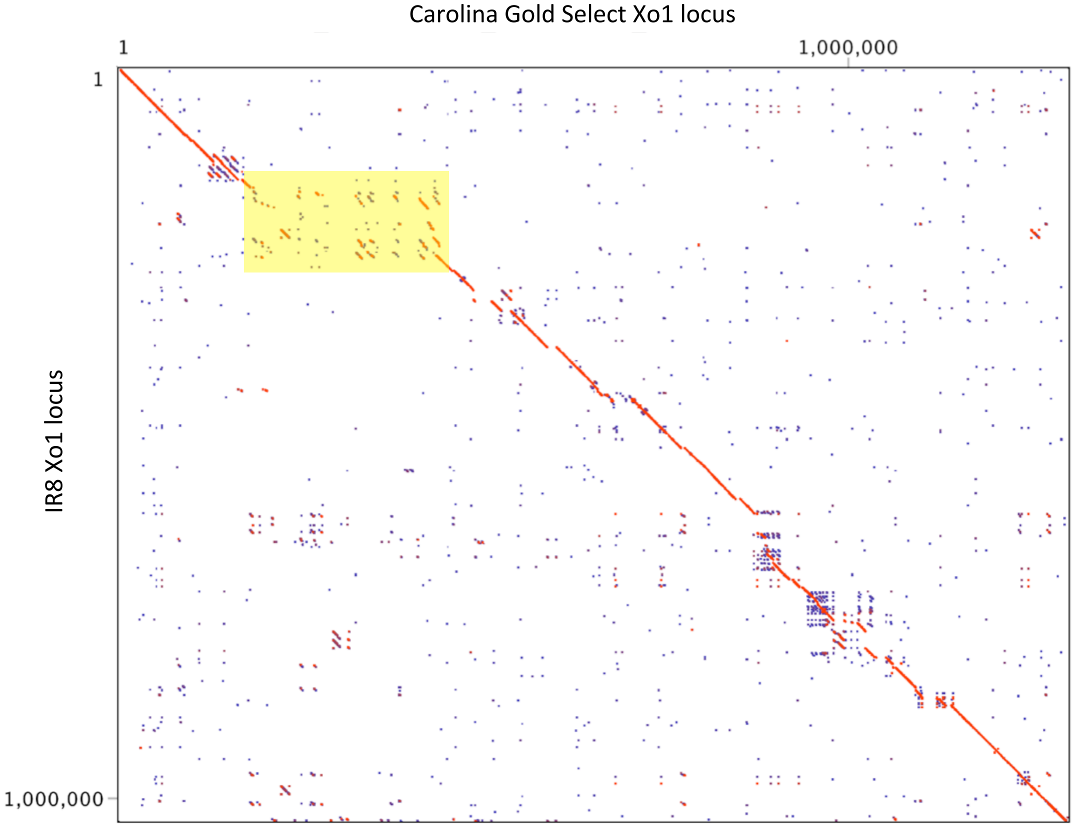

Dotplot comparison of the Xo1 region of Carolina Gold Select (chr4 22729801..24027920) and indica cultivar IR8 (chr4 31657596..32688920). The yellow box highlights the insertion that is present in Carolina Gold Select and absent in IR8.
