# Supplementary material for: Genome assembly and characterization of a complex zfBED-NLR gene-containing disease resistance locus in Carolina Gold Select rice with Nanopore sequencing
Source: PLoS Genet. 2020 Jan 27;16(1):e1008571. doi: 10.1371/journal.pgen.1008571 (PMC7004385; doi:10.1371/journal.pgen.1008571)
Supplement: S2 Fig — Maximum likelihood tree of 5,083 NB-ARC domain amino acid sequences detected by NLR-Annotator in representative Oryzeae genomes. Tree includes known rice R-genes and three wheat zfBED-NLRs. NB-ARC domains with stop codons were included in the tree. Xo1 clade I and II are highlighted with orange and purple branches respectively. NB-ARC amino acid sequences are available in Supplemental S3 Table. Tree file is available at iTOL– http://itol.eml.de/shared/acr242. (PDF) [file pgen.1008571.s013.pdf]

S2 Figure

Maximum likelihood tree of NB-ARC domain from Oryzeae NLRs

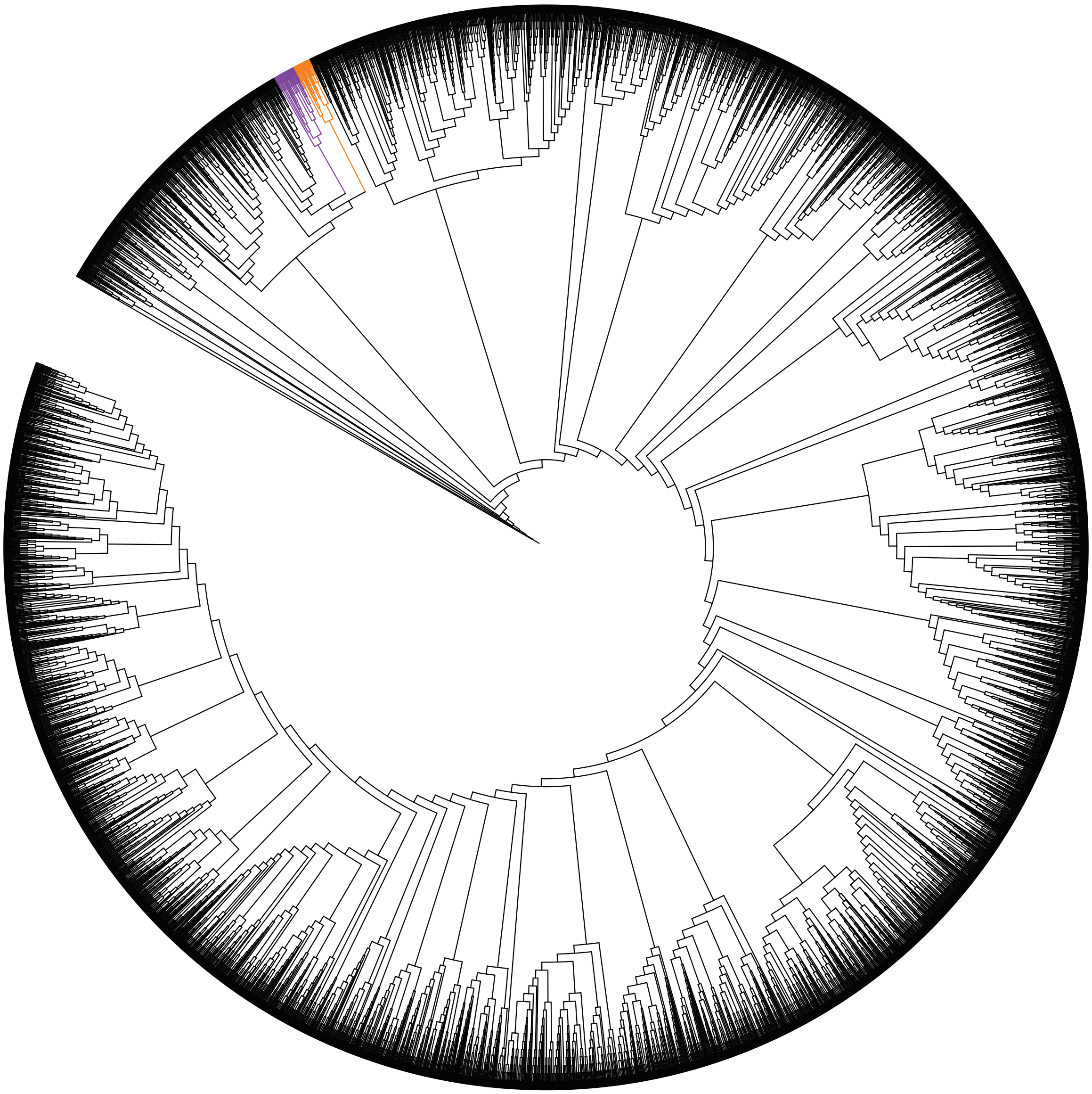

Maximum likelihood tree of 5083 NB-ARC domain amino acid sequences detected by NLR-Annotator in representative Oryzeae genomes. Tree includes known rice R-genes and three wheat zfBED-NLRs. NB-ARC domains with stop codons were included in the tree. Xo1 clade I and II are highlighted with orange and purple branches respectively. NB-ARC amino acid sequences are available in Supplemental table S3. Tree file is available at iTOL – http://itol.eml.de/shared/acr242.
