# Supplementary material for: Genome assembly and characterization of a complex zfBED-NLR gene-containing disease resistance locus in Carolina Gold Select rice with Nanopore sequencing
Source: PLoS Genet. 2020 Jan 27;16(1):e1008571. doi: 10.1371/journal.pgen.1008571 (PMC7004385; doi:10.1371/journal.pgen.1008571)
Supplement: S3 Fig — Maximum likelihood tree of zfBED domain nucleotide sequences from Xo1 clade I and II NLRs. Branches with bootstrap support greater than 80 percent are indicated with pink squares. Interactive tree available at http://itol.embl.de/shared/acr242. (PDF) [file pgen.1008571.s014.pdf]

S3 Figure
Maximum likelihood tree of NB-ARC domain from Oryzeae NLRs

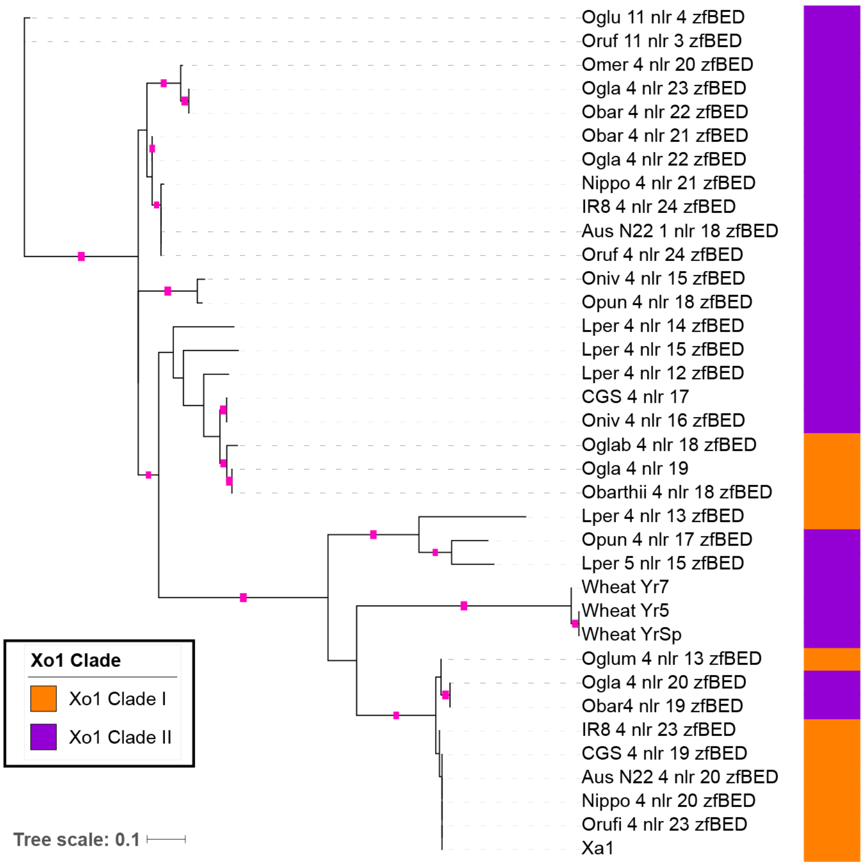

Maximum likelihood tree of zfBED domain nucleotide sequences from Xo1 clade I and II NLRs. Branches with bootstrap support greater than 80 percent are indicated with pink squares. Interactive tree available at http://itol.embl.de/shared/acr242.
